# THE IMPORTANCE OF LIFELONG LEARNING FOR AVOIDING EXCLUSION—POLICY FRAMEWORKS AND EXPERIENCES OF OLDER WORKERS

**DOI:** 10.1093/geroni/igad104.1789

**Published:** 2023-12-21

**Authors:** Nehle Penning, Rachel Crossdale, Monika Reichert

**Affiliations:** TU Dortmund University, Dortmund, Nordrhein-Westfalen, Germany; The University of Sheffield, Sheffield, England, United Kingdom; TU Dortmund University, Dortmund, Nordrhein-Westfalen, Germany

## Abstract

Qualification requirements in the labour market in Europe have increased across the board. Increasing provision and accessibility of lifelong learning is central to promoting employability among older workers. However, older workers are more likely to lack formal and to hold out of date qualifications. Sweden, Poland, Germany and the UK, despite differences in regime and political approach to lifelong learning, have unequal distribution of access and opportunity. This paper examines how older workers in the four countries assess the role of lifelong learning throughout working life as well as what links to national policies can be identified. The analysis is based on 100 semi-structured and problem-centred interviews in the four countries with older workers (age 55 and older) of different backgrounds (gender, educational level, sector of employment, ethnicity). Content analysis according to Kuckartz (2018) was used as method of analysis. The interview data shows that lifelong learning plays an important role for older workers in the prevention and/or management of exclusion risks throughout working life. However, the potential of lifelong learning for EWL is limited by barriers at the company and regulative level and differences between the public and private sector are noticeable. In late working life, in particular financial constraints as well as ageism lead to inequalities in relation to lifelong learning opportunities. In all four nations a higher priority need to be given to an integral support of lifelong learning throughout the life-course as well as age stereotypes need to be dismantled for a successful EWL agenda.

